# Plasma ApoE elevations are associated with NAFLD: The PREVEND Study

**DOI:** 10.1371/journal.pone.0220659

**Published:** 2019-08-06

**Authors:** Eline H. van den Berg, James P. Corsetti, Stephan J. L. Bakker, Robin P. F. Dullaart

**Affiliations:** 1 Department of Endocrinology, University of Groningen, University Medical Center Groningen, Groningen, The Netherlands; 2 Department of Gastroenterology and Hepatology, University of Groningen, University Medical Center Groningen, Groningen, The Netherlands; 3 Department of Pathology and Laboratory Medicine, University of Rochester School of Medicine and Dentistry, Rochester, New York, United States of America; 4 Department of Nephrology, University of Groningen, University Medical Center Groningen, Groningen, The Netherlands; University of Colorado Denver School of Medicine, UNITED STATES

## Abstract

Non-alcoholic fatty liver disease (NAFLD) is featured by increased plasma very low density lipoproteins (VLDL). The extent to which plasma apolipoprotein E (ApoE) levels are elevated in NAFLD is unclear. We determined whether plasma ApoE is elevated in subjects with suspected NAFLD. Plasma ApoE and genotypes were determined in 6,762 participants of the Prevention of Renal and Vascular End-Stage Disease (PREVEND) cohort. A Fatty Liver Index (FLI) ≥ 60 was used as a proxy of NAFLD. A total of 1,834 participants had a FLI ≥ 60, which coincided with increased triglycerides, non-HDL cholesterol, ApoB and ApoE (all *P*<0.001). In multivariable linear regression analysis, plasma ApoE levels were positively associated with an elevated FLI when taking account of ApoE genotypes and other clinical and laboratory covariates (fully adjusted model: β = 0.201, *P*<0.001). Stratified analysis for ApoE genotypes (ApoE ε3ε3 homozygotes, ApoE ε2 carriers, and ApoE ε3ε4 and ε4ε4 carriers combined), also showed positive associations of plasma ApoE levels with an elevated FLI in each group (all *P*<0.001). In conclusion, it is suggested that NAFLD is characterized by increased plasma ApoE levels, even when taking account of the various ApoE genotypes. Increased plasma ApoE may contribute to altered VLDL metabolism and to increased atherosclerosis susceptibility in NAFLD.

## Introduction

Non-alcoholic fatty liver disease (NAFLD) is characterized by hepatic steatosis in the absence of alcohol abuse, and is emerging as the most common cause of chronic liver disease. The spectrum of NAFLD ranges from simple steatosis to non-alcoholic steatohepatitis (NASH), fibrosis and eventually cirrhosis [1–3]. NAFLD is considered to be the liver manifestation of the metabolic syndrome (MetS) and coincides with an increased risk for the development of type 2 diabetes mellitus (T2D) [1,3–5]. Furthermore, NAFLD is characterized by plasma lipoprotein abnormalities, including elevations in apoliporotein (Apo)B-containing lipoproteins and decreased levels of high density lipoprotein (HDL) cholesterol [6–8], which predispose to atherosclerotic cardiovascular disease (CVD) [9,10]. Hepatic fat accumulation is regarded as the driving force of enhanced production of very low-density lipoproteins (VLDL) by the liver, resulting in an increased plasma concentration of large VLDL particles and consequently in higher triglycerides [11–14].

Apolipoprotein E (ApoE) is a 34 kDA protein comprised of 299 amino acids [15]. The liver is a major source of circulating ApoE [16,17]. ApoE plays an important role in the production of VLDL as well as in their clearance by facilitating the hepatic uptake of triglyceride-rich lipoproteins (TRL) via ApoE-mediated binding of TRL remnants to hepatic lipoprotein receptors [18–21]. ApoE may also contribute to inhibition of inflammation and oxidative stress [22]. In plasma, ApoE is strongly associated with VLDL and hence with triglycerides [23–26]. Higher total plasma ApoE levels are also observed in MetS [27], and may confer increased CVD risk, though unlikely independent of ApoB [28]. Additionally, ApoE was observed to be associated with CVD in women with elevated HDL cholesterol in combination with high C-reactive protein (CRP) levels [29,30].

The *APOE* gene is located on chromosome 19 and has 3 common alleles (ε2, ε3 and ε4) leading to six ApoE genotypes; three homozygotes (ε2ε2, ε3ε3 and ε4ε4) and three heterozygotes (ε2ε4, ε2ε3, ε3ε4) [31,32]. ApoE polymorphisms account in part for the variability of plasma ApoE with the lowest levels in ApoE ε4 carriers [33], and are known to affect lipoprotein receptor-binding abilities. A direct effect of ApoE genotypes on total cholesterol and low density lipoprotein cholesterol (LDL-C) is well established [33,34]. Furthermore, a meta-analysis showed a linear relationship between ApoE genotypes and coronary risk [35], emphasizing the importance of ApoE gene variation for atherosclerosis development.

Conversely, ApoE could also be involved in the development of hepatic fat accumulation. In diet-induced NAFLD murine models, ApoE deficiency was suggested to retard hepatic lipid deposition [36]. In ApoE deficient mice, a Western high-fat cholesterol-rich diet accelerates the formation of NASH with fibrosis [37], but the association of plasma ApoE levels with NAFLD has not been previously tested in humans. Additionally, several small-scale studies reported on the possible association of ApoE genotypes with NAFLD but showed conflicting results [38–43].

Since ApoE may affect VLDL metabolism, it is plausible to hypothesize that NAFLD is featured by higher plasma ApoE levels. In the absence of previous reports on the possible impact of NAFLD on plasma ApoE, we initiated the present study to examine whether increased plasma ApoE levels relate to prevalent NAFLD. We also questioned whether such an association is affected by ApoE genotypes. To this end we carried out a cross-sectional analysis among 6,762 subjects participating in the Prevention of REnal and Vascular ENd-stage Disease (PREVEND) cohort study, comprising a large and well-characterized population from the north of the Netherlands.

## Materials and methods

### Study population

The study was performed among participants of the Prevention of REnal and Vascular ENd-stage Disease (PREVEND) cohort study [44,45]. PREVEND is a large prospective general population-based study, that was initiated to investigate cardiovascular and renal disease with a focus on albuminuria. All inhabitants (28 to 75 years old) of Groningen, the Netherlands were send a questionnaire on demographics and cardiovascular morbidity and were asked to supply an early morning urine specimen. Pregnant women, type 1 diabetic subjects and type 2 diabetic subjects using insulin were not allowed to participate. All participants with a urinary albumin concentration ≥ 10 mg/L were invited to our clinic together with randomly selected subjects with a urinary albumin concentration < 10 mg/L. The initial study population of the PREVEND study comprised 8,592 subjects who completed the total study screening program. The PREVEND study was approved by the Medical Ethics Committee of the University Medical Center Groningen and is performed in accordance with Declaration of Helsinki guidelines [44,45]. All participants gave written informed consent.

For the present study, we excluded subjects in which data on plasma ApoE, clinical and biochemical variables to calculate the Fatty Liver Index (FLI), a proxy of NAFLD, were not available, leaving a study population of 6,762 participants.

### Measurements and definitions

Measurements and definitions are reported as described in detail previously [46]. Body mass index (BMI) was calculated as weight (kg) divided by height squared (meter). Waist circumference was measured as the smallest girth between rib cage and iliac crest. The waist/hip ratio was determined as the waist circumference divided by the largest girth between waist and thigh [44]. Blood pressure was measured using an automatic device. T2D was defined as a fasting glucose ≥ 7.0 mmol/L, a random glucose ≥ 11.1 mmol/L, self-report of a physician diagnosis or the use of glucose lowering drugs. Alcohol consumption was recorded with one alcoholic drink being assumed to contain 10 grams of alcohol. Smoking was categorized into current and never/former smokers. Past cardiovascular history included: hospitalization for myocardial ischemia, obstructive coronary artery disease or revascularization procedures. Urinary albumin excretion (UAE) was measured as described in two 24-hour urine collections and the results were averaged for analysis [44]. Estimated glomerular filtration rate (eGFR) was calculated applying the combined creatinine cystatin C-based Chronic Kidney Disease Epidemiology Collaboration equation [47]. Information on medication use was combined with information from a pharmacy-dispensing registry, which has complete information on drug usage of > 95% of subjects in the PREVEND study. Venous blood samples were drawn after an overnight fast while the participants had rested for 15 minutes.

For the diagnosis of suspected NAFLD, the algorithm of the Fatty Liver Index (FLI) was used [48]. The FLI was calculated according to the following formula [48]:

$$\begin{array}{c} {}[e\;(0.953\;x\;loge\;(triglycerides+0.139\;x\;BMI+0.718\;x\;loge\;\left(GGT\right)+0.053\;x\;waist\\ circumference–15.745)/[1+e\;(0.953\;x\;loge\;\left(triglycerides\right)\;+\;0.139\;x\;BMI\;+\;0.718\;x\;loge\;\left(GGT\right)+\\ 0.053\;x\;waist\;circumference–15.745)]\;x\;100, \end{array}$$

*where GGT is gamma-glutamyltransferase*.

The optimal cut-off value for the FLI is documented to be 60 with an accuracy of 84%, a sensitivity of 61% and a specificity of 86% for detecting suspected NAFLD as determined by ultrasonography [48]. FLI ≥ 60 was therefore used as proxy of NAFLD. The FLI is currently considered as one of the best-validated steatosis scores for larger scale screening studies [49]. Alternatively, we used the Hepatic Steatosis Index (HSI) which has thus far predominantly been used in Asian populations [50]. The HSI is defined as follows:

HSI=8×ALT/ASTratio+BMI(+2,ifdiabetes;+2,iffemale),

*where ALT is alanine aminotransferase and AST is aspartate aminotransferase*.

The cut-off value of the HSI for detecting suspected NAFLD is 36 [50]. In these equations, BMI is expressed in kg/m^2^, triglycerides are expressed in mmol/L, and GGT, ALT and AST are expressed in U/L.

The MetS was defined according to the revised National Cholesterol Education Program Adult Treatment Panel (NCEP-ATP) III criteria [51]. Participants were categorized with MetS when at least three out of five of the following criteria were present: waist circumference > 102 cm for men and > 88 cm for women; plasma triglycerides ≥ 1.7 mmol/L; HDL cholesterol < 1.0 mmol/L for men and < 1.3 mmol/L for women; hypertension (blood pressure ≥ 130/85 mm Hg or the use of antihypertensive medication); hyperglycemia (fasting glucose ≥ 5.6 mmol/L or the use of glucose lowering drugs).

### Laboratory methods

Laboratory methods are reported as described in detail previously [46]. Heparinized plasma and serum samples were obtained by centrifugation at 1400x *g* for 15 min at 4°C. Plasma and serum samples were stored at -80°C until analysis. Glucose was measured directly after blood collection. Plasma total cholesterol, triglycerides, HDL cholesterol, Apo A-I, ApoB and ApoE were measured as previously described [28–30,44,45]. ApoE genotyping was performed as described previously [52]. Non-HDL cholesterol was calculated as the difference between total cholesterol and HDL cholesterol. LDL cholesterol was calculated by the Friedewald formula if triglycerides were < 4.5 mmol/L [53]. Serum ALT and AST were measured using the standardized kinetic method with pyridoxal phosphate activation (Roche Modular P; Roche Diagnostics, Mannheim, Germany). Serum GGT was assayed by an enzymatic colorimetric method (Roche Modular P, Roche Diagnostics, Mannheim, Germany). Standardization of ALT, AST and GGT was performed according to International Federation of Clinical Chemistry guidelines [54–56]. hsCRP was assayed by nephelometry. Serum creatinine was measured by an enzymatic method on a RocheModular analyzer (Roche Diagnostics, Mannheim, Germany). Serum cystatin C was measured by Gentian Cystatin C Immunoassay (Gentian AS, Moss, Norway) on a Modular analyzer (Roche Diagnostics). Urinary albumin was measured by nephelometry (Dade Behring Diagnostic, Marburg, Germany).

### Statistical analysis

IBM SPSS software (version 23.0 Armonk, NY: IBM Corp) was used for data analysis. Results are expressed as mean ± standard deviation (SD), median with interquartile range (IQR) or as numbers (percentages). Normality of distribution was assessed and checked for skewness. Between group differences in variables were determined by unpaired T-tests for normally distributed variables, Mann-Whitney U test for non-normally distributed variables or by Chi-square tests for categorical variables where appropriate. Multivariable linear regression analyses were carried out to disclose the independent associations of ApoE levels with an elevated FLI and HSI when taking account of clinical covariates and laboratory parameters, including ApoE genotype. Stratified analyses were additionally performed in ApoE ε3 homozygotes, ApoE ε2 carriers (ε2ε2, ε2ε3 and ε2ε4 genotypes combined) and ApoE ε3ε4 and ε4ε4 carriers combined. Two-sided *P*-values < 0.05 were considered significant.

## Results

### Clinical and laboratory characteristics of the study population

The study population consisted of 6,762 subjects of whom 1,834 (27.1%) were classified with a FLI ≥ 60, as proxy of NAFLD. Table 1 shows the clinical characteristics and laboratory data of the participants according to the FLI categorization. Relatively more men had a FLI ≥ 60 (men 68.8% vs. women 31.2%). Subjects with a FLI ≥ 60 were more likely to be classified with MetS and T2D, and had a cardiovascular history more frequently. Antihypertensive medication, and glucose and lipid lowering drugs were taken more frequently in subjects with a FLI ≥ 60. Alcohol consumption ≥ 10 gram/day was recorded in subjects with an elevated FLI more frequently, but cigarette smoking was not different between subjects with and without an elevated FLI. BMI, waist circumference, the waist/hip ratio, systolic and diastolic blood pressure, plasma glucose, hsCRP, ALT, AST, GGT, UAE, total cholesterol, non-HDL cholesterol, LDL cholesterol and triglycerides were higher in subjects with an elevated FLI, but eGFR and HDL cholesterol were lower in subjects with an elevated FLI (Table 1). ApoE and ApoB were higher, whereas ApoA-I was lower in subjects with an elevated FLI (*P*<0.001 for each). ApoE genotype distribution was not significantly different between subjects (multinomial Chi-square, *P* = 0.19). Both in subjects with and without an elevated FLI, ApoE genotype ε3ε3 was most frequent, followed by a descending frequency of ε3ε4, ε2ε3, ε2ε4, ε4ε4 and ε2ε2 ApoE genotypes (Table 1).

**Table 1:** Clinical and laboratory characteristics including plasma apolipoprotein E in 4,928 subjects with a Fatty Liver Index (FLI) < 60 and 1,834 subjects with a FLI ≥ 60.

	FLI < 60 n = 4,928 (72.9)	FLI ≥ 60 n = 1,834 (27.1)	*P*
**Age** (years), mean ± SD	47.8 ± 12.5	55.2 ± 11.6	<0.001
**Sex** (men/women), n (%)	2,100 (42.6) / 2,828 (57.4)	1,261 (68.8) / 573 (31.2)	<0.001
**T2D**, n (%)	86 (1.7)	158 (8.6)	<0.001
**MetS**, n (%)	414 (8.4)	1,142 (62.3)	<0.001
**History of cardiovascular disease**, n (%)	178 (3.6)	167 (9.1)	<0.001
**Current smokers**, n (%)	1,661 (33.7)	602 (32.8)	0.54
**Alcohol ≥10 g/day**, n (%)	1,172 (23.8)	552 (30.1)	<0.001
**Antihypertensive medication**, n (%)	509 (10.3)	532 (29.0)	<0.001
**Glucose lowering drugs**, n (%)	54 (1.1)	69 (3.8)	<0.001
**Lipid lowering drugs**, n (%)	212 (4.3)	208 (11.3)	<0.001
**Systolic blood pressure** (mm Hg), mean ± SD	125 ± 19	141 ± 20	<0.001
**Diastolic blood pressure** (mm Hg), mean ± SD	72 ± 9	79 ± 9	<0.001
**BMI** (kg/m^2^), mean ± SD	24.4 ± 2.90	30.5 ± 3.92	<0.001
**Waist circumference**, mean ± SD	82.9 ± 9.6	102.9 ± 8.9	<0.001
**Waist/hip ratio**, mean ± SD	0.85 ± 0.08	0.96 ± 0.08	<0.001
**Glucose** (mmol/L), mean ± SD	4.63 ± 0.83	5.35 ± 1.48	<0.001
**hsCRP** (mg/L), median (IQR)	0.97 (0.44–2.32)	2.35 (1.19–4.78)	<0.001
**ALT** (U/L), median (IQR)	18 (14–24)	28 (20–39)	<0.001
**AST** (U/L), median (IQR)	23 (20–27)	27 (23–32)	<0.001
**GGT** (U/L), median (IQR)	20 (14–28)	41 (29–65)	<0.001
**eGFR** (ml/min/1.73 m^2^), mean ± SD	97.0 ± 16.5	88.9 ± 17.8	<0.001
**UAE** (mg/24 hr), median (IQR)	8.4 (6.0–14.0)	14.0 (8.0–32.3)	<0.001
**Total cholesterol** (mmol/L), mean ± SD	5.46 ± 1.09	6.08 ± 1.08	<0.001
**Non-HDL cholesterol** (mmol/L), mean ± SD	4.04 ± 1.13	4.98 ± 1.09	<0.001
**LDL cholesterol** (mmol/L), mean ± SD	3.54 ± 1.03	4.07 ± 1.02	<0.001
**HDL cholesterol** (mmol/L), mean ± SD	1.42 ± 0.40	1.11 ± 0.30	<0.001
**Triglycerides** (mmol/L), median (IQR)	1.00 (0.76–1.33)	1.84 (1.36–2.48)	<0.001
**ApoA-I** (g/L), mean ± SD	1.420 ± 0.302	1.304 ± 0.272	<0.001
**ApoB** (g/L), mean ± SD	0.972 ± 0.284	1.185 ± 0.307	<0.001
**ApoE** (g/L), mean ± SD	0.036 ± 0.012	0.045 ± 0.017	<0.001
**ApoE genotype**			0.19
**ApoE genotype ε2ε2**, n (%)	29 (0.6)	19 (1.0)	
**ApoE genotype ε2ε3**, n (%)	566 (11.5)	233 (12.7)	
**ApoE genotype ε2ε4**, n (%)	116 (2.4)	47 (2.6)	
**ApoE genotype ε3ε3**, n (%)	2,633 (53.4)	944 (51.5)	
**ApoE genotype ε3ε4**, n (%)	1,163 (23.6)	431 (23.5)	
**ApoE genotype ε4ε4**, n (%)	124 (2.5)	39 (2.1)	

Data are given in number with percentages (%), mean ± standard deviation (SD) for normally distributed data or median with interquartile ranges (IQR) for non-normally distributed data. Abbreviations: ALT, alanine aminotransferase; ApoA-I, apolipoprotein A-I; ApoB, apolipoprotein B; ApoE, apolipoprotein E; AST, aspartate aminotransferase; AU, arbitrary units; BMI, body mass index; FLI, Fatty Liver Index; eGFR, estimated glomerular filtration rate; GGT, gamma-glutamyltransferase; HDL, high density lipoproteins; hsCRP, high sensitivity C-reactive protein; LDL, low density lipoproteins; MetS, metabolic syndrome; T2D, type 2 diabetes mellitus; UAE, urinary albumin excretion. LDL cholesterol was calculated by the Friedewald formula in 4,903 subjects with a FLI < 60 and in 1,735 subjects with a FLI ≥ 60.

### Independent relationships of plasma ApoE with an elevated FLI and HSI

Multivariable linear regression analyses were subsequently performed in order to establish the extent to which plasma ApoE was associated with an elevated FLI (Table 2). In age- and sex-adjusted analysis a positive association of plasma ApoE with an elevated FLI was found (Table 2, Model 1, β = 0.299, *P*<0.001). This association of plasma ApoE with an elevated FLI remained present after adjustment for T2D, MetS, alcohol intake, current smoking and the various ApoE genotypes (Table 2, Model 2, β = 0.206, *P*<0.001). When further adjusted for eGFR, UAE, a cardiovascular disease history and use of antihypertensive medication and glucose and lipid lowering drugs, plasma ApoE remained associated with an elevated FLI (Table 2, Model 3, β = 0.201, *P*<0.001). Plasma ApoE was also associated with an elevated FLI in alternative analysis adjusted for glucose, non-HDL cholesterol and HDL cholesterol (Table 2, Model 4, β = 0.181, *P*<0.001) or for glucose, ApoB and ApoA-1 (Table 2, Model 5, β = 0.204, *P*<0.001). Furthermore, these analyses demonstrated higher ApoE levels in the context of MetS, higher non-HDL cholesterol, HDL cholesterol, ApoB and ApoA-1 as well as higher ApoE levels in ε2 carriers and lower ApoE levels in ε4 carriers (Table 2, Models 2–5). In an alternative analysis with an elevated HSI instead of an elevated FLI, a similar independent positive association of plasma ApoE with an elevated HSI was found (Table 3, all models, *P*<0.001). Moreover, in analysis with HSI and triglycerides as independent variables, ApoE was associated with an elevated HSI (β = 0.029, *P* = 0.003) independent of triglycerides. Additionally, analysis with comparison of an elevated FLI ≥ 60 with FLI < 30 (S1 Table) and elevated HSI > 36 with HSI < 30 (S2 Table), as a lower cut-off for excluding suspected NAFLD, showed even stronger associations with plasma ApoE.

**Table 2:** Multivariable regression analysis demonstrating the positive association of plasma apolipoprotein E with an elevated Fatty Liver Index (FLI) (≥ 60) after adjustment for clinical and laboratory covariates in 6,762 subjects.

	Model 1		Model 2		Model 3		Model 4		Model 5	
	β	*P*	β	*P*	β	*P*	β	*P*	β	*P*
**Age**	0.124	< 0.001	0.095	< 0.001	0.097	< 0.001	-0.025	0.057	0.019	0.146
**Sex** (men vs. women)	-0.030	0.010	0.004	0.785	0.005	0.730	-0.011	0.408	-0.006	0.681
**FLI** ≥ 60 vs. < 60	0.299	< 0.001	0.206	< 0.001	0.201	< 0.001	0.181	< 0.001	0.204	< 0.001
**T2D** (yes/no)			0.002	0.907	0.071	< 0.001				
**MetS** (yes/no)			0.176	< 0.001	0.182	< 0.001				
**Glucose**							0.061	< 0.001	0.080	< 0.001
**Non-HDL cholesterol** (mmol/L)							0.472	< 0.001		
**HDL cholesterol** (mmol/L)							0.094	< 0.001		
**ApoB** (g/L)									0.358	< 0.001
**ApoA-1** (g/L)									0.089	< 0.001
**Alcoholic intake** (≥10 g/day)			-0.004	0.792	-0.005	0.725	-0.017	0.189	-0.023	0.070
**Current smoking** (yes/no)			0.027	0.043	0.027	0.050	-0.007	0.579	-0.009	0.468
**ApoE genotype ε2ε2 vs. ε3ε3**			0.295	< 0.001	0.296	< 0.001	0.320	< 0.001	0.343	< 0.001
**ApoE genotype ε2ε3 vs. ε3ε3**			0.223	< 0.001	0.224	< 0.001	0.278	< 0.001	0.272	< 0.001
**ApoE genotype ε2ε4 vs. ε3ε3**			0.100	< 0.001	0.099	< 0.001	0.108	< 0.001	0.114	< 0.001
**ApoE genotype ε3ε4 vs. ε3ε3**			-0.070	< 0.001	-0.070	< 0.001	-0.095	< 0.001	-0.085	< 0.001
**ApoE genotype ε4ε4 vs. ε3ε3**			-0.070	< 0.001	-0.069	< 0.001	-0.089	< 0.001	-0.085	< 0.001
**eGFR** (ml/min/1.73 m^2^)					-0.012	0.492				
**UAE** (mg/24 hr)					0.042	0.002				
**History of cardiovascular disease**					-0.026	0.065				
**Use of antihypertensive medication**					-0.020	0.186				
**Use of glucose lowering drugs**					-0.094	< 0.001				
**Use of lipid lowering drugs**					-0.003	0.817				

β: standardized regression coefficients. ApoA-1, apolipoprotein A-1, ApoB, apolipoprotein B; ApoE, apolipoprotein E; eGFR, estimated glomerular filtration rate; FLI, Fatty Liver Index; HDL, high density lipoproteins; MetS, metabolic syndrome; T2D, type 2 diabetes mellitus, UAE; urinary albumin excretion. The ApoE ε3ε3 genotype was used as reference category for the various ApoE genotypes.

**Model 1**: adjusted for age and sex.

**Model 2**: adjusted for age, sex, T2D, MetS, alcoholic intake, current smoking and ApoE genotype.

**Model 3**: adjusted for age, sex, T2D, MetS, alcoholic intake, current smoking, ApoE genotype, history of cardiovascular disease, eGFR, UAE and use of antihypertensive medication, glucose lowering and lipid lowering drugs.

**Model 4**: adjusted for age, sex, glucose, non-HDL cholesterol, HDL cholesterol, alcoholic intake, current smoking and ApoE genotype.

**Model 5**: adjusted for age, sex, glucose, ApoB, ApoA-1, alcoholic intake, current smoking and ApoE genotype.

**Table 3:** Multivariable regression analysis demonstrating the positive association of plasma apolipoprotein E with an elevated Hepatic Steatosis Index (HSI) (> 36) after adjustment for clinical and laboratory covariates in 6,762 subjects.

	Model 1		Model 2		Model 3		Model 4		Model 5	
	β	*P*	β	*P*	β	*P*	β	*P*	β	*P*
**Age**	0.168	< 0.001	0.105	< 0.001	0.105	< 0.001	-0.012	0.373	0.034	0.012
**Sex** (men vs. women)	0.029	0.013	0.039	0.005	0.039	0.004	0.009	0.509	0.023	0.096
**HSI >** 36 vs. ≤ 36	0.176	< 0.001	0.084	< 0.001	0.080	< 0.001	0.074	< 0.001	0.099	< 0.001
**T2D** (yes/no)			-0.007	0.607	0.068	0.001				
**MetS** (yes/no)			0.253	< 0.001	0.257	< 0.001				
**Glucose**							0.075	< 0.001	0.096	< 0.001
**Non-HDL cholesterol** (mmol/L)							0.500	< 0.001		
**HDL cholesterol** (mmol/L)							0.071	< 0.001		
**ApoB** (g/L)									0.394	< 0.001
**ApoA-1** (g/L)									0.076	< 0.001
**Alcoholic intake** (≥10 g/day)			0.003	0.853	0.001	0.916	-0.009	0.469	-0.017	0.202
**Current smoking** (yes/no)			0.038	0.006	0.036	0.009	-0.003	0.841	-0.002	0.894
**ApoE genotype ε2ε2 vs. ε3ε3**			0.296	< 0.001	0.297	< 0.001	0.323	< 0.001	0.349	< 0.001
**ApoE genotype ε2ε3 vs. ε3ε3**			0.225	< 0.001	0.226	< 0.001	0.284	< 0.001	0.280	< 0.001
**ApoE genotype ε2ε4 vs. ε3ε3**			0.102	< 0.001	0.100	< 0.001	0.109	< 0.001	0.116	< 0.001
**ApoE genotype ε3ε4 vs. ε3ε3**			-0.069	< 0.001	-0.069	< 0.001	-0.096	< 0.001	-0.085	< 0.001
**ApoE genotype ε4ε4 vs. ε3ε3**			-0.067	< 0.001	-0.066	< 0.001	-0.088	< 0.001	-0.084	< 0.001
**eGFR** (ml/min/1.73 m^2^)					-0.016	0.373				
**UAE** (mg/24 hr)					0.047	0.001				
**History of cardiovascular disease**					-0.027	0.060				
**Use of antihypertensive medication**					-0.015	0.310				
**Use of glucose lowering drugs**					-0.101	< 0.001				
**Use of lipid lowering drugs**					-0.007	0.637				

β: standardized regression coefficients. ApoA-1, apolipoprotein A-1, ApoB, apolipoprotein B; ApoE, apolipoprotein E; eGFR, estimated glomerular filtration rate; HDL, high density lipoproteins, HSI, Hepatic Steatosis Index; MetS, metabolic syndrome; T2D, type 2 diabetes mellitus, UAE; urinary albumin excretion. The ApoE ε3ε3 genotype was used as reference category for the various ApoE genotypes.

**Model 1**: adjusted for age and sex.

**Model 2**: adjusted for age, sex, T2D, MetS, alcoholic intake, current smoking and ApoE genotype.

**Model 3**: adjusted for age, sex, T2D, MetS, alcoholic intake, current smoking, ApoE genotype, history of cardiovascular disease, eGFR, UAE and use of antihypertensive medication, glucose lowering and lipid lowering drugs.

**Model 4**: adjusted for age, sex, glucose, non-HDL cholesterol, HDL cholesterol, alcoholic intake, current smoking and ApoE genotype.

**Model 5**: adjusted for age, sex, glucose, ApoB, ApoA-1, alcoholic intake, current smoking and ApoE genotype.

A sensitivity analysis with exclusion of subjects with alcoholic intake ≥ 10 g/day, a positive cardiovascular history, impaired eGFR (< 60 mL/min/1.73 m^2^), elevated UAE (> 30 mg/24 hr), use of antihypertensive drugs, glucose and lipid lowering drugs, leaving 3,501 subjects for analysis, also demonstrated a positive association of plasma ApoE levels with an elevated FLI when taking account of T2D, MetS, smoking and ApoE genotype (Table 4, all models *P*<0.001).

**Table 4:** Multivariable regression analysis demonstrating the positive association of plasma apolipoprotein E with an elevated Fatty Liver Index (FLI) (≥ 60) after adjustment for clinical and laboratory covariates in 3,501 subjects, excluding subjects with alcoholic intake ≥ 10 g/day, a positive cardiovascular history, impaired estimated glomerular filtration rate (< 60 mL/min/1.73 m^2^), elevated urinary albumin excretion (> 30 mg/24 hr), use of antihypertensive drugs, glucose lowering drugs and lipid lowering drugs.

	Model 1		Model 2		Model 3	
	β	*P*	β	*P*	β	*P*
**Age**	0.167	< 0.001	0.156	< 0.001	0.146	< 0.001
**Sex** (men vs. women)	-0.032	0.047	-0.018	0.354	0.011	0.517
**FLI** ≥ 60 vs. < 60	0.283	< 0.001	0.197	< 0.001	0.191	< 0.001
**T2D** (yes/no)			0.029	0.137	0.028	0.102
**MetS** (yes/no)			0.154	< 0.001	0.153	< 0.001
**Current smoking** (yes/no)			0.009	0.653	0.013	0.430
**ApoE genotype ε2ε2 vs. ε3ε3**					0.359	< 0.001
**ApoE genotype ε2ε3 vs. ε3ε3**					0.248	< 0.001
**ApoE genotype ε2ε4 vs. ε3ε3**					0.107	< 0.001
**ApoE genotype ε3ε4 vs. ε3ε3**					-0.100	< 0.001
**ApoE genotype ε4ε4 vs. ε3ε3**					-0.088	< 0.001

β: standardized regression coefficients. ApoE, apolipoprotein E; FLI, Fatty Liver Index; MetS, metabolic syndrome; T2D, type 2 diabetes mellitus. The ApoE ε3ε3 genotype was used as reference category for the various ApoE genotypes.

**Model 1**: adjusted for age and sex.

**Model 2**: adjusted for age, sex, T2D, MetS and current smoking.

**Model 3**: adjusted for age, sex, T2D, MetS, current smoking and ApoE genotype.

Stratified analyses according to ApoE genotype showed significant associations of ApoE with an elevated FLI in ApoE ε3ε3 homozygotes (S3 Table), ApoE ε2 carriers (ε2ε2, ε2ε3 and ε2ε4 genotypes combined) (S4 Table) and a combination of genotype ApoE ε3ε4 and ε4ε4 (S5 Table) (*P*<0.001 for each genotype group and *P*<0.001 in all models).

## Discussion

The present large-scale cross-sectional study in a predominantly Caucasian population demonstrates to the best of our knowledge for the first time that plasma ApoE levels, a well-recognized determinant of VLDL and hence of triglyceride metabolism, are positively associated with NAFLD. In our study, we used an elevated FLI [48], and in alternative analyses an elevated HSI [50], as proxies of NAFLD, in line with international guidelines, which recommend using biomarkers in order to categorize subjects with probable NAFLD in large-scale studies [49]. In multivariable linear regression analyses, plasma ApoE levels remained positively associated with an elevated FLI when taking account of the various ApoE genotypes, T2D, MetS, glucose, non-HDL cholesterol, ApoB and other relevant covariates. Analyses with an elevated HSI instead of an elevated FLI iterated these findings. Furthermore, in a sensitivity analysis, excluding subjects with a cardiovascular history, impaired eGFR, elevated UAE and use of medication, as well as in analyses stratified for ApoE genotypes (ApoE ε3ε3 homozygotes, ApoE ε2 carriers and ApoE ε3ε4 and ε4ε4 combined) an independent positive association of plasma ApoE levels with an elevated FLI was also demonstrated. Taken together, the present report thus suggests that higher plasma ApoE levels are increased in the context of an elevated FLI, as a proxy of NAFLD.

Imbalances between fatty acid influx, utilization and triglyceride synthesis leads to hepatic steatosis by accumulation of triglycerides and cholesteryl esters in hepatocytes [11–14,57]. Also the impact of hepatic fat accumulation on enhanced VLDL secretion is well established, as evidenced by higher VLDL and ApoB production rates in the context of hepatic fat accumulation [11–14]. The liver is a major source of ApoE, which controls intracellular lipid metabolism and VLDL assembly [57]. In murine models of ApoE deficiency and ApoE overexpression as well as in *in vitro* experiments, ApoE has been identified as an important regulator of hepatic VLDL assembly and secretion [57–59]. A link between ApoE and VLDL secretion has also been demonstrated in humans [57,60–62]. Furthermore, ApoE also plays an important role in VLDL clearance with the liver being the major site for the metabolism of ApoE-containing lipoproteins [14,18,63,64]. In an ApoE genotype-dependent fashion, ApoE has key functions in the binding and uptake of circulating lipoproteins which arise largely from its role in promoting clearance of TRL from the circulation involving the LDL receptor, the low density lipoprotein receptor-related protein 1 and heparan sulphate proteoglycans [63,64]. Additionally, ApoE may also affect TRL lipolysis [64]. The current report makes it plausible that a higher plasma ApoE level is a feature of NAFLD, conceivably by affecting VLDL metabolism although the precise mechanisms responsible for the association of higher plasma ApoE with NAFLD remain to be established.

Previous small-scale studies on the possible association of ApoE genotype variation with NAFLD showed conflicting results: no association of the different ApoE alleles with NAFLD [39], a possible protective effect of the ApoE ε4 allele against NAFLD [38,43], a possible protective effect of the ApoE ε2 allele and ApoE ε2ε3 genotype in non-obese NAFLD subjects [40], a higher risk for advanced fibrosis in ApoE ε4 carriers compared with ε3 carriers [41] and an association of increased NASH formation in the ApoE ε3ε3 genotype [42]. In our study, multivariable linear regression analysis demonstrated that plasma ApoE was higher in ApoE ε2 allele carriers and lower in ApoE ε4 allele carriers compared with ApoE ε3 allele carriers as expected [33]. No significant difference was found in the ApoE genotype distribution between individuals with and without suspected NAFLD, suggesting that ApoE gene variation as such has no major impact on NAFLD development. The differences between the present study and previous reports could in part be explained by the small number of study participants in these earlier studies [39–43], different approaches for NAFLD diagnosis and differences in ethnical background i.e. Dutch-Caucasian *vs*. Turkish, Italian, Polish or Korean populations. Earlier reports demonstrated a lower hepatic VLDL ApoB production rate in ApoE ε2 homozygotes and ApoE ε4 homozygotes compared with ApoE ε3 homozygotes [60], and a lower VLDL ApoB production rate in ApoE ε4 carriers compared with ApoE ε3 homozygotes [62]. In the current study, plasma ApoE was also positively associated with an elevated FLI in analyses stratified for different ApoE genotype groups (ε3ε3 homozygotes, ε2 and ε4 carriers).

It is plausible to postulate that the association of plasma ApoE with NAFLD as shown in this report could influence the alleged effect of NAFLD on atherosclerosis susceptibility. Plasma ApoE levels predict incident CVD which is probably explained at least in part by the association of ApoE levels with atherogenic TRL [28,65,66]. In addition, ApoE has a potential role in dysfunctional transformation of HDL [29,30]. ApoE exerts anti-oxidative properties as well [22], and stabilizes the activity of paraoxonase-1 (PON-1), an HDL associated enzyme with anti-oxidative properties [67,68]. Serum PON-1 activity is indeed positively correlated with ApoE, but this relationship was found to be abolished in MetS, probably consequent to MetS-associated abnormalities in HDL [26].

Several strengths, limitations and methodological aspects of the present study need to be discerned. First, we performed a cross-sectional analysis. For this reason cause-effect relationships cannot be established with certainty, nor can we exclude the possibility of reversed causation. Thus while our study was analyzed with plasma ApoE as independent variable, it is also possible that circulating ApoE levels as such may represent a determinant of hepatic fat accumulation. Second, an elevated FLI was chosen as a proxy of suspected NAFLD. The FLI is considered to have sufficient accuracy for NAFLD assessment, and its use is in line with international guidelines to apply biomarker scores in order to characterize NAFLD in larger-sized cohorts and seems to perform best in European subjects, which is probably related to the ethnical difference in fat distribution [48,49]. Moreover, the positive association of plasma ApoE levels with suspected NAFLD, was confirmed by using the HSI as an alternative algorithm for NAFLD categorization [50], where it should be noted that the HSI has only been validated in a Korean population with a non-Caucasian background [50]. Also, analysis with a lower cut-off for excluding suspected NAFLD (FLI < 30 and HSI < 30), showed even stronger associations with plasma ApoE. Performing liver ultrasound or liver biopsy for the diagnosis of NAFLD, was not feasible in the PREVEND cohort study, which recruited individuals from the general population. Third, we could not differentiate between simple hepatic steatosis and hepatic fibrosis; therefore, no relationship of hepatic fibrosis with plasma ApoE levels could be established. Fourth, to preclude interactions with the FLI in the statistical analysis, variables making part of the FLI equation (i.e. triglycerides) were excluded as independent variables in multivariable analyses. Instead, we used i) metabolic syndrome categorization, ii) non-HDL cholesterol or iii) alternatively plasma ApoB as measures of ApoB-containing lipoproteins in subsidiary analyses. Fifth, we adjusted for HDL cholesterol as well, reasoning that these lipoproteins also carry ApoE [27]. Finally, the proportion of subjects using alcohol in excess of 30 gram per day in the PREVEND cohort is low, i.e. about 5.2% [69]. We adjusted for alcohol consumption in all analyses, and ApoE was unrelated to alcohol consumption. Also, the association of an elevated FLI with plasma ApoE remained present in analysis in which we excluded subjects with alcoholic intake ≥ 10 g/day. Finally, people with microalbuminuria preferentially participated in the PREVEND cohort. Therefore, we adjusted for eGFR and UAE in multivariable regression analysis and carried out a sensitivity analysis excluding subjects with impaired eGFR and elevated UAE. Reassuringly these analyses showed similar positive and independent associations of plasma ApoE levels with suspected NAFLD.

In conclusion, this study shows that suspected NAFLD is characterized by increased plasma ApoE levels, which conceivably contribute to altered VLDL metabolism in NAFLD.

## Supporting information

S1 Table Multivariable regression analysis demonstrating the positive association of plasma apolipoprotein E with an elevated Fatty Liver Index (FLI) ≥ 60 in 1,834 subjects compared with FLI < 30 in 3,270 subjects after adjustment for clinical and laboratory covariates. (DOCX)

S2 Table Multivariable regression analysis demonstrating the positive association of plasma apolipoprotein E with an elevated Hepatic Steatosis Index (HSI) (> 36) in 1,862 subjects compared with HSI < 30 in 1,465 subjects after adjustment for clinical and laboratory covariates. (DOCX)

S3 Table Multivariable regression analysis demonstrating the positive association of plasma apolipoprotein E with an elevated Fatty Liver Index (FLI) (≥ 60) after adjustment for clinical and laboratory covariates in 3,577 subjects with apolipoprotein E genotype ε3ε3. (DOCX)

S4 Table Multivariable regression analysis demonstrating the positive association of plasma apolipoprotein E with an elevated Fatty Liver Index (FLI) (≥ 60) after adjustment for clinical and laboratory covariates in 1,010 subjects with apolipoprotein E ε2 carriers (ε2ε2, ε2ε3 and ε2ε4 genotypes combined). (DOCX)

S5 Table Multivariable regression analysis demonstrating the positive association of plasma apolipoprotein E with an elevated Fatty Liver Index (FLI) (≥ 60) after adjustment for clinical and laboratory covariates in 1,757 subjects with apolipoprotein E genotype ε3ε4 and ε4ε4. (DOCX)

S1 Data Database. (SAV)
