# Supplementary material for: Plasma ApoE elevations are associated with NAFLD: The PREVEND Study
Source: PLoS One. 2019 Aug 6;14(8):e0220659. doi: 10.1371/journal.pone.0220659 (PMC6684074; doi:10.1371/journal.pone.0220659)
Supplement: S1 Table — (DOCX) [file pone.0220659.s001.docx]

**S1 Table**. Multivariable regression analysis demonstrating the positive association of plasma apolipoprotein E with an elevated Fatty Liver Index (FLI) ≥ 60 in 1,834 subjects compared with FLI < 30 in 3,270 subjects after adjustment for clinical and laboratory covariates.

|  | **Model 1** |  | **Model 2** |  | **Model 3** |  | **Model 4** |  | **Model 5** |  |
| --- | --- | --- | --- | --- | --- | --- | --- | --- | --- | --- |
|  | β | *P* | β | *P* | β | *P* | β | *P* | β | *P* |
| **Age** | 0.088 | < 0.001 | 0.070 | < 0.001 | 0.077 | < 0.001 | -0.047 | 0.002 | -0.004 | 0.796 |
| **Sex** (men vs. women) | -0.041 | 0.003 | -0.011 | 0.500 | -0.011 | 0.497 | -0.010 | 0.498 | -0.004 | 0.790 |
| **FLI** ≥ 60 vs. < 30 | 0.356 | < 0.001 | 0.237 | < 0.001 | 0.233 | < 0.001 | 0.188 | < 0.001 | 0.228 | < 0.001 |
| **T2D** (yes/no) |  |  | 0.000 | 0.990 | 0.068 | 0.003 |  |  |  |  |
| **MetS** (yes/no) |  |  | 0.176 | < 0.001 | 0.180 | < 0.001 |  |  |  |  |
| **Glucose** |  |  |  |  |  |  | 0.060 | < 0.001 | 0.081 | < 0.001 |
| **Non-HDL cholesterol** (mmol/L) |  |  |  |  |  |  | 0.480 | < 0.001 |  |  |
| **HDL cholesterol** (mmol/L) |  |  |  |  |  |  | 0.108 | < 0.001 |  |  |
| **ApoB** (g/L) |  |  |  |  |  |  |  |  | 0.330 | < 0.001 |
| **ApoA-1** (g/L) |  |  |  |  |  |  |  |  | 0.093 | < 0.001 |
| **Alcoholic intake** (≥10 g/day) |  |  | 0.003 | 0.833 | 0.002 | 0.904 | -0.011 | 0.437 | -0.014 | 0.331 |
| **Current smoking** (yes/no) |  |  | 0.032 | 0.040 | 0.031 | 0.047 | -0.003 | 0.811 | -0.002 | 0.903 |
| **ApoE genotype ε2ε2 vs. ε3ε3** |  |  | 0.292 | < 0.001 | 0.292 | < 0.001 | 0.309 | < 0.001 | 0.331 | < 0.001 |
| **ApoE genotype ε2ε3 vs. ε3ε3** |  |  | 0.206 | < 0.001 | 0.206 | < 0.001 | 0.259 | < 0.001 | 0.247 | < 0.001 |
| **ApoE genotype ε2ε4 vs. ε3ε3** |  |  | 0.098 | < 0.001 | 0.098 | < 0.001 | 0.108 | < 0.001 | 0.112 | < 0.001 |
| **ApoE genotype ε3ε4 vs. ε3ε3** |  |  | -0.065 | < 0.001 | -0.064 | < 0.001 | -0.091 | < 0.001 | -0.079 | < 0.001 |
| **ApoE genotype ε4ε4 vs. ε3ε3** |  |  | -0.063 | < 0.001 | -0.064 | < 0.001 | -0.086 | < 0.001 | -0.078 | < 0.001 |
| **eGFR** (ml/min/1.73 m^2^) |  |  |  |  | -0.004 | 0.822 |  |  |  |  |
| **UAE** (mg/24 hr) |  |  |  |  | 0.046 | 0.002 |  |  |  |  |
| **History of cardiovascular disease** |  |  |  |  | -0.024 | 0.140 |  |  |  |  |
| **Use of antihypertensive medication** |  |  |  |  | -0.022 | 0.196 |  |  |  |  |
| **Use of glucose lowering drugs** |  |  |  |  | -0.090 | < 0.001 |  |  |  |  |
| **Use of lipid lowering drugs** |  |  |  |  | -0.007 | 0.677 |  |  |  |  |

*β: standardized regression coefficients. ApoA-1, apolipoprotein A-1, ApoB, apolipoprotein B; ApoE, apolipoprotein E; eGFR, estimated glomerular filtration rate; FLI, Fatty Liver Index; HDL, high density lipoproteins; MetS, metabolic syndrome; T2D, type 2 diabetes mellitus, UAE; urinary albumin excretion. The ApoE ε3ε3 genotype was used as reference category for the various ApoE genotypes.*

***Model 1****: adjusted for age and sex.*

***Model 2****: adjusted for age, sex, T2D, MetS, alcoholic intake, current smoking and ApoE genotype.*

***Model 3****: adjusted for age, sex, T2D, MetS, alcoholic intake, current smoking, ApoE genotype, history of cardiovascular disease, eGFR, UAE and use of antihypertensive medication, glucose lowering and lipid lowering drugs.*

***Model 4****: adjusted for age, sex, glucose, non-HDL cholesterol, HDL cholesterol, alcoholic intake, current smoking and ApoE genotype.*

***Model 5****: adjusted for age, sex, glucose, ApoB, ApoA-1, alcoholic intake, current smoking and ApoE genotype.*
