# Supplementary material for: Plasma ApoE elevations are associated with NAFLD: The PREVEND Study
Source: PLoS One. 2019 Aug 6;14(8):e0220659. doi: 10.1371/journal.pone.0220659 (PMC6684074; doi:10.1371/journal.pone.0220659)
Supplement: S2 Table — (DOCX) [file pone.0220659.s002.docx]

**S2 Table**. Multivariable regression analysis demonstrating the positive association of plasma apolipoprotein E with an elevated Hepatic Steatosis Index (HSI) (> 36) in 1,862 subjects compared with HSI < 30 in 1,465 subjects after adjustment for clinical and laboratory covariates.

|  | **Model 1** |  | **Model 2** |  | **Model 3** |  | **Model 4** |  | **Model 5** |  |
| --- | --- | --- | --- | --- | --- | --- | --- | --- | --- | --- |
|  | β | *P* | β | *P* | β | *P* | β | *P* | β | *P* |
| **Age** | 0.110 | < 0.001 | 0.060 | 0.003 | 0.068 | 0.009 | -0.040 | 0.039 | 0.004 | 0.852 |
| **Sex** (men vs. women) | 0.042 | 0.011 | 0.053 | 0.006 | 0.054 | 0.006 | 0.019 | 0.317 | 0.044 | 0.024 |
| **HSI >** 36 vs. < 30 | 0.243 | < 0.001 | 0.130 | < 0.001 | 0.128 | < 0.001 | 0.077 | < 0.001 | 0.120 | < 0.001 |
| **T2D** (yes/no) |  |  | 0.004 | 0.859 | 0.070 | 0.012 |  |  |  |  |
| **MetS** (yes/no) |  |  | 0.245 | < 0.001 | 0.254 | < 0.001 |  |  |  |  |
| **Glucose** |  |  |  |  |  |  | 0.074 | < 0.001 | 0.094 | < 0.001 |
| **Non-HDL cholesterol** (mmol/L) |  |  |  |  |  |  | 0.508 | < 0.001 |  |  |
| **HDL cholesterol** (mmol/L) |  |  |  |  |  |  | 0.063 | 0.003 |  |  |
| **ApoB** (g/L) |  |  |  |  |  |  |  |  | 0.381 | < 0.001 |
| **ApoA-1** (g/L) |  |  |  |  |  |  |  |  | 0.064 | 0.001 |
| **Alcoholic intake** (≥10 g/day) |  |  | 0.029 | 0.143 | 0.024 | 0.221 | 0.019 | 0.279 | 0.011 | 0.548 |
| **Current smoking** (yes/no) |  |  | 0.021 | 0.283 | 0.020 | 0.310 | -0.023 | 0.197 | -0.021 | 0.264 |
| **ApoE genotype ε2ε2 vs. ε3ε3** |  |  | 0.302 | < 0.001 | 0.301 | < 0.001 | 0.317 | < 0.001 | 0.348 | < 0.001 |
| **ApoE genotype ε2ε3 vs. ε3ε3** |  |  | 0.208 | < 0.001 | 0.205 | < 0.001 | 0.264 | < 0.001 | 0.262 | < 0.001 |
| **ApoE genotype ε2ε4 vs. ε3ε3** |  |  | 0.132 | < 0.001 | 0.133 | < 0.001 | 0.139 | < 0.001 | 0.144 | < 0.001 |
| **ApoE genotype ε3ε4 vs. ε3ε3** |  |  | -0.074 | < 0.001 | -0.073 | < 0.001 | -0.091 | < 0.001 | -0.077 | < 0.001 |
| **ApoE genotype ε4ε4 vs. ε3ε3** |  |  | -0.049 | 0.010 | -0.048 | 0.012 | -0.072 | < 0.001 | -0.070 | < 0.001 |
| **eGFR** (ml/min/1.73 m^2^) |  |  |  |  | -0.005 | 0.838 |  |  |  |  |
| **UAE** (mg/24 hr) |  |  |  |  | 0.001 | 0.963 |  |  |  |  |
| **History of cardiovascular disease** |  |  |  |  | -0.012 | 0.560 |  |  |  |  |
| **Use of antihypertensive medication** |  |  |  |  | -0.021 | 0.314 |  |  |  |  |
| **Use of glucose lowering drugs** |  |  |  |  | -0.092 | < 0.001 |  |  |  |  |
| **Use of lipid lowering drugs** |  |  |  |  | -0.018 | 0.364 |  |  |  |  |

*β: standardized regression coefficients. ApoA-1, apolipoprotein A-1, ApoB, apolipoprotein B; ApoE, apolipoprotein E; eGFR, estimated glomerular filtration rate; HDL, high density lipoproteins, HSI, Hepatic Steatosis Index; MetS, metabolic syndrome; T2D, type 2 diabetes mellitus, UAE; urinary albumin excretion. The ApoE ε3ε3 genotype was used as reference category for the various ApoE genotypes.*

***Model 1****: adjusted for age and sex.*

***Model 2****: adjusted for age, sex, T2D, MetS, alcoholic intake, current smoking and ApoE genotype.*

***Model 3****: adjusted for age, sex, T2D, MetS, alcoholic intake, current smoking, ApoE genotype, history of cardiovascular disease, eGFR, UAE and use of antihypertensive medication, glucose lowering and lipid lowering drugs.*

***Model 4****: adjusted for age, sex, glucose, non-HDL cholesterol, HDL cholesterol, alcoholic intake, current smoking and ApoE genotype.*

***Model 5****: adjusted for age, sex, glucose, ApoB, ApoA-1, alcoholic intake, current smoking and ApoE genotype.*
