# Supplementary material for: Plasma ApoE elevations are associated with NAFLD: The PREVEND Study
Source: PLoS One. 2019 Aug 6;14(8):e0220659. doi: 10.1371/journal.pone.0220659 (PMC6684074; doi:10.1371/journal.pone.0220659)
Supplement: S3 Table — (DOCX) [file pone.0220659.s003.docx]

**S3 Table**. Multivariable regression analysis demonstrating the positive association of plasma apolipoprotein E with an elevated Fatty Liver Index (FLI) (≥ 60) after adjustment for clinical and laboratory covariates in 3,577 subjects with apolipoprotein E genotype ε3ε3.

|  | **Model 1** |  | **Model 2** |  | **Model 3** |  |
| --- | --- | --- | --- | --- | --- | --- |
|  | β | *P* | β | *P* | β | *P* |
| **Age** | 0.184 | < 0.001 | 0.152 | < 0.001 | 0.190 | < 0.001 |
| **Sex** (men vs. women) | -0.064 | < 0.001 | -0.032 | 0.114 | -0.042 | 0.041 |
| **FLI** ≥ 60 vs. < 60 | 0.282 | < 0.001 | 0.209 | < 0.001 | 0.303 | < 0.001 |
| **T2D** (yes/no) |  |  | 0.058 | 0.004 |  |  |
| **MetS** (yes/no) |  |  | 0.171 | < 0.001 |  |  |
| **Alcoholic intake** (≥10 g/day) |  |  | -0.034 | 0.092 | -0.034 | 0.096 |
| **Current smoking** (yes/no) |  |  | 0.016 | 0.434 | 0.014 | 0.487 |
| **eGFR** (ml/min/1.73 m^2^) |  |  |  |  | -0.003 | 0.923 |
| **UAE** (mg/24 hr) |  |  |  |  | 0.100 | < 0.001 |
| **History of cardiovascular disease** |  |  |  |  | -0.044 | 0.040 |
| **Use of antihypertensive medication** |  |  |  |  | -0.007 | 0.765 |
| **Use of glucose lowering drugs** |  |  |  |  | -0.004 | 0.832 |
| **Use of lipid lowering drugs** |  |  |  |  | 0.023 | 0.280 |

β: standardized regression coefficients. ApoE, apolipoprotein E; eGFR, estimated glomerular filtration rate; FLI, Fatty Liver Index; MetS, metabolic syndrome; T2D, type 2 diabetes mellitus; UAE, urinary albumin excretion,

**Model 1**: adjusted for age and sex.

**Model 2**: adjusted for age, sex, T2D, MetS, alcoholic intake and current smoking.

**Model 3**: adjusted for age, sex, alcoholic intake, current smoking, history of cardiovascular disease, eGFR, UAE and use of antihypertensive medication, glucose lowering and lipid lowering drugs.
