# Supplementary material for: Plasma ApoE elevations are associated with NAFLD: The PREVEND Study
Source: PLoS One. 2019 Aug 6;14(8):e0220659. doi: 10.1371/journal.pone.0220659 (PMC6684074; doi:10.1371/journal.pone.0220659)
Supplement: S4 Table — (DOCX) [file pone.0220659.s004.docx]

**S4 Table**. Multivariable regression analysis demonstrating the positive association of plasma apolipoprotein E with an elevated Fatty Liver Index (FLI) (≥ 60) after adjustment for clinical and laboratory covariates in 1,010 subjects with apolipoprotein E ε2 carriers (ε2ε2, ε2ε3 and ε2ε4 genotypes combined).

|  | **Model 1** |  | **Model 2** |  | **Model 3** |  |
| --- | --- | --- | --- | --- | --- | --- |
|  | β | *P* | β | *P* | β | *P* |
| **Age** | 0.116 | < 0.001 | 0.096 | 0.015 | 0.123 | 0.013 |
| **Sex** (men vs. women) | -0.023 | 0.442 | -0.004 | 0.909 | -0.001 | 0.979 |
| **FLI** ≥ 60 vs. < 60 | 0.321 | < 0.001 | 0.177 | < 0.001 | 0.312 | < 0.001 |
| **T2D** (yes/no) |  |  | -0.096 | 0.013 |  |  |
| **MetS** (yes/no) |  |  | 0.261 | < 0.001 |  |  |
| **Alcoholic intake** (≥10 g/day) |  |  | -0.011 | 0.776 | -0.020 | 0.611 |
| **Current smoking** (yes/no) |  |  | 0.056 | 0.142 | 0.053 | 0.179 |
| **eGFR** (ml/min/1.73 m^2^) |  |  |  |  | -0.048 | 0.314 |
| **UAE** (mg/24 hr) |  |  |  |  | -0.051 | 0.193 |
| **History of cardiovascular disease** |  |  |  |  | 0.007 | 0.857 |
| **Use of antihypertensive medication** |  |  |  |  | 0.011 | 0.798 |
| **Use of glucose lowering drugs** |  |  |  |  | -0.053 | 0.187 |
| **Use of lipid lowering drugs** |  |  |  |  | -0.045 | 0.282 |

β: standardized regression coefficients. ApoE, apolipoprotein E; eGFR, estimated glomerular filtration rate; FLI, Fatty Liver Index; MetS, metabolic syndrome; T2D, type 2 diabetes mellitus, UAE; urinary albumin excretion. ApoE ε2 carriers include genotypes ε2ε2, ε2ε3 and ε2ε4.

**Model 1**: adjusted for age and sex.

**Model 2**: adjusted for age, sex, T2D, MetS, alcoholic intake and current smoking.

**Model 3**: adjusted for age, sex, alcoholic intake, current smoking, history of cardiovascular disease, eGFR, UAE and use of antihypertensive medication, glucose lowering drugs and lipid lowering drugs.
