# Supplementary material for: Plasma ApoE elevations are associated with NAFLD: The PREVEND Study
Source: PLoS One. 2019 Aug 6;14(8):e0220659. doi: 10.1371/journal.pone.0220659 (PMC6684074; doi:10.1371/journal.pone.0220659)
Supplement: S5 Table — (DOCX) [file pone.0220659.s005.docx]

**S5 Table**. Multivariable regression analysis demonstrating the positive association of plasma apolipoprotein E with an elevated Fatty Liver Index (FLI) (≥ 60) after adjustment for clinical and laboratory covariates in 1,757 subjects with apolipoprotein E genotype ε3ε4 and ε4ε4.

|  | **Model 1** |  | **Model 2** |  | **Model 3** |  |
| --- | --- | --- | --- | --- | --- | --- |
|  | β | *P* | β | *P* | β | *P* |
| **Age** | 0.057 | 0.011 | 0.036 | 0.203 | 0.054 | 0.148 |
| **Sex** (men vs. women) | 0.045 | 0.044 | 0.054 | 0.062 | 0.044 | 0.133 |
| **FLI** ≥ 60 vs. < 60 | 0.374 | < 0.001 | 0.271 | < 0.001 | 0.370 | < 0.001 |
| **T2D** (yes/no) |  |  | -0.030 | 0.287 |  |  |
| **MetS** (yes/no) |  |  | 0.181 | < 0.001 |  |  |
| **Alcoholic intake** (≥10 g/day) |  |  | 0.041 | 0.153 | 0.033 | 0.249 |
| **Current smoking** (yes/no) |  |  | 0.046 | 0.099 | 0.051 | 0.073 |
| **eGFR** (ml/min/1.73 m^2^) |  |  |  |  | 0.004 | 0.906 |
| **UAE** (mg/24 hr) |  |  |  |  | -0.032 | 0.259 |
| **History of cardiovascular disease** |  |  |  |  | -0.008 | 0.789 |
| **Use of antihypertensive medication** |  |  |  |  | 0.036 | 0.239 |
| **Use of glucose lowering drugs** |  |  |  |  | -0.037 | 0.197 |
| **Use of lipid lowering drugs** |  |  |  |  | 0.033 | 0.266 |

β: standardized regression coefficients. ApoE, apolipoprotein E; eGFR, estimated glomerular filtration rate; FLI, Fatty Liver Index; MetS, metabolic syndrome; T2D, type 2 diabetes mellitus, UAE; urinary albumin excretion.

**Model 1**: adjusted for age and sex.

**Model 2**: adjusted for age, sex, T2D, MetS, alcoholic intake and current smoking.

**Model 3**: adjusted for age, sex, alcoholic intake, current smoking, history of cardiovascular disease, eGFR, UAE and use of antihypertensive medication, glucose lowering drugs and lipid lowering drugs.
